# Impact of Intramedullary Implants on Metallic Element Homeostasis in Children with Forearm Fractures

**DOI:** 10.3390/jcm14217829

**Published:** 2025-11-04

**Authors:** Kacper Sowa, Anna Danielewicz, Magdalena Wójciak, Jan Sawicki, Sławomir Dresler, Katarzyna Warda, Michał Latalski, Ireneusz Sowa

**Affiliations:** 1 Department of Paediatric Orthopaedics, Medical University of Lublin, 20-059 Lublin, Polandkatarzyna.m.warda@gmail.com (K.W.); 2 Department of Analytical Chemistry, Medical University of Lublin, 20-059 Lublin, Poland; magdalena.wojciak@umlub.edu.pl (M.W.); jan.sawicki@umlub.edu.pl (J.S.); slawomir.dresler@umlub.edu.pl (S.D.)

**Keywords:** pediatric fractures, intramedullary fixation, titanium implants, metallic element, titanium, biocompatibility, inductively coupled plasma optical emission

## Abstract

**Background/Objectives**: Childhood is marked by frequent musculoskeletal injuries, with fractures representing a major cause of pediatric trauma admissions. Unstable long-bone fractures often require surgical stabilization, commonly achieved using elastic stable intramedullary nailing (ESIN). Although this method ensures effective fixation and early mobilization, concerns remain regarding potential metal ion release in growing children. This study aimed to assess changes in calcium, magnesium, copper, zinc, titanium, and aluminum concentrations in blood and material from the medullary cavity of forearm fractures following intramedullary fixation. **Methods**: A prospective study was conducted on 40 patients aged 4–15 years treated with ESIN at the University Children’s Hospital in Lublin. Peripheral blood and material from the medullary cavity were collected before implantation and at implant removal. Elemental concentrations were determined using high-resolution ICP-OES, and statistical analyses included paired comparisons, delta values, and multivariate methods. **Results**: No significant systemic changes were found for calcium, magnesium, copper, zinc, or aluminum. A modest but significant increase in blood titanium levels was observed after treatment (*p* = 0.0075), especially in patients with two rods. Multivariate analysis confirmed overall stability of elemental profiles, with titanium contributing most strongly to post-treatment variation. **Conclusions**: Intramedullary titanium fixation in children does not significantly disrupt systemic mineral homeostasis. The slight increase in circulating titanium reflects implant exposure rather than toxicity, supporting the safety of ESIN, although continued monitoring of metallic elements may be warranted.

## 1. Introduction

Childhood is a period characterized by intense physical activity combined with a limited sense of risk and caution in everyday life. Consequently, this stage of life is accompanied by a high incidence of injuries, particularly of the musculoskeletal system.

Among these injuries, fractures are the most common, accounting for approximately 25% of admissions to pediatric trauma and orthopedic surgery wards [1]. Unstable fractures or those with significant angular deformity usually require surgical management, most commonly internal fixation. Surgical treatment is also favored when rapid mobilization and recovery are required to minimize disruption to daily activities and allow children to return to school as quickly as possible.

Advances in orthopedic surgical techniques and implant technology have expanded the range of available treatment options. The choice of treatment must always be tailored to the patient’s age, the fracture type, and the anticipated balance of risks and benefits [2,3]. In cases of unstable fractures of long bones with open growth plates, elastic stable intramedullary nailing (ESIN) has become the preferred method [4,5]. This technique, performed with titanium or stainless-steel elastic nails of 1.5–4 mm diameter depending on the medullary canal, provides stable fixation with minimal invasiveness, short operative time, low complication rates, and allows early mobilization without prolonged casting [3,5,6,7]. Plate-and-screw fixations are used much less frequently in children and mainly after skeletal maturity [5,7,8,9,10].

Despite their benefits, intramedullary implants are subject to corrosion, mechanical stress, and possible fracture. The surrounding biological environment, rich in chloride ions and oxygen, can promote electrochemical processes, particularly when part of the implant lies in soft tissues. Additionally, biofilm formation on the implant surface may accelerate biocorrosion [11]. Furthermore, the choice of an intramedullary implant (IMN) itself, as opposed to periosteal materials like plates, is associated with a specific local inflammatory response within the medullary cavity, which also constitutes a key element of the implant-tissue interaction [12]. The oxide layers covering metallic materials play a key protective role, but corrosion and mechanical wear can still lead to the release of metal ions into the surrounding bone and soft tissues. These ions may remain local or enter systemic circulation, potentially eliciting cellular responses, hypersensitivity reactions, or even systemic effects. In some cases, they may bind to host proteins or macromolecules, which has been hypothesized to contribute to carcinogenesis [13].

Given these concerns, understanding the local and systemic impact of orthopedic implants in children is essential.

The aim of this study was to analyze and evaluate changes in the concentrations of selected elements in peripheral blood and material from the medullary cavity in pediatric patients undergoing surgical treatment of forearm fractures using intramedullary fixation. Specifically, we sought to determine: (I) whether the levels of selected elements change after the implantation of internal fixations, (II) whether the number of implants influences the degree of change, (III) whether intramedullary fixations affect systemic metal ion levels, and (IV) whether there is a relationship between the patient’s age and observed changes.

## 2. Materials and Methods

### 2.1. Participants of the Study

**Figure 1:**
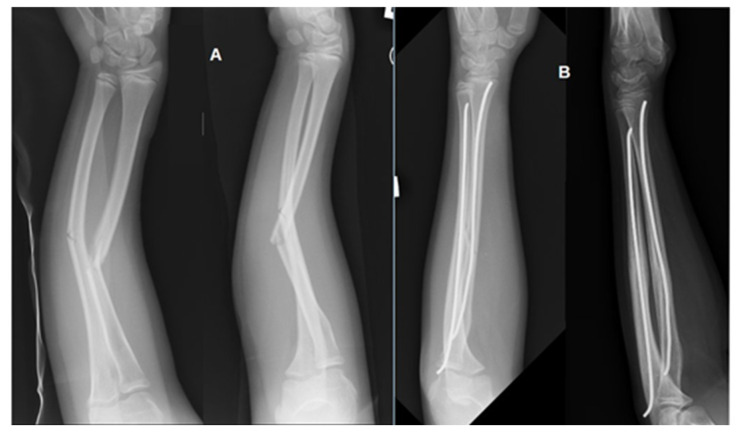
Pre (**A**) and postoperative (**B**) anteroposterior (AP) and lateral (LAT) radiographs of a forearm fracture in a patient from the study group. The fracture of both the radius and ulna was stabilized using the Elastic Stable Intramedullary Nailing (ESIN) technique with two titanium nails.

A prospective study was conducted at the Pediatric Orthopedic Clinic of the University Children’s Hospital in Lublin. The study population comprised patients who underwent surgical treatment for forearm fractures using internal fixation with elastic intramedullary nails between 2018 and 2020. Inclusion criteria were defined as follows: age between 4 and 15 years, fracture of one or both forearm bones, surgical management with elastic intramedullary nailing, including both implantation and subsequent removal procedures, and absence of other internal fixations (Figure 1).

The exclusion criteria included chronic diseases (e.g., liver or endocrine disorders, diabetes) as well as treatment with antiepileptic drugs or corticosteroids. In addition, written informed consent from the children’s parents or legal guardians was required for participation in the study. Out of 976 cases reviewed, 40 patients fulfilled all inclusion criteria and were included in the final analysis. A detailed summary of participants’ demographic characteristics is presented in Table 1.

The study was approved by the Bioethics Committee of the Medical University of Lublin (consent no. KE-0254/105/2015; date of approval: 30 April 2015). Written informed consent was obtained from all participants and their parents.

### 2.2. Research Material

For each patient, a peripheral blood sample was collected prior to surgical procedures during routine testing, with a minimum volume of 3 mL, into a sterile polypropylene tube containing EDTA and free of trace elements (S-Monovette, SARSTEDT AG & Co. KG, Nümbrecht, Germany). During the surgical procedure, material was obtained directly from the medullary cavity of the implanted bone. After skin incision, tissue dissection, bone exposure, and hemostasis, a needle attached to a syringe was inserted into the medullary cavity through a hole created several centimeters deep (at least 2.5 cm). The sample was drawn directly from the cavity, whereas any fluid freely flowing from the wound was not collected for analysis. Additional post-operative samples from the medullary cavity and peripheral blood were collected at the time of implant removal, using the same opening created for implant placement. The mean interval between implantation and implant removal for the study cohort was 8.35 months (median: 9 months).

### 2.3. Elemental Analysis

To prepare the sample for analysis, 0.3–0.5 mL of the sample was placed in a vessel, and 1.5 mL of 63% HNO_3_ Suprapur^®^ (Sigma–Aldrich, Darmstadt, Germany) and 5 mL of deionised water were added. The vessels were sealed, placed in a DigiPREP MS heating block (SCP Science, Baie D’Urfe, QC, Canada), and the digestion process was carried out at a temperature of 130 °C for 60 min. After cooling to room temperature, samples were diluted with deionized water to a final volume of 25 mL. Quantitative analysis was performed using high–resolution inductively coupled plasma–optical emission spectrometry (ICP-OES) PlasmaQuant^®^ PQ 9000 Elite (Analytik Jena, Jena, Germany). The wavelengths 315,887, 327,396, 285,213, 334,941, 206,200, and 396,152 nm were used for the determination of Ca, Cu, Mg, Ti, Zn, and Al, respectively. The reading for Ca and Mg was performed in attenuated radial mode, while the reading for other elements was performed in axial mode. The device operating parameters were as follows: RF power—1300 W, gas flows: plasma 12.0 L/min, auxiliary 0.5 L/min, nebulizer 0.6 L/min, sample flow rate 2 mL/min. The limits of quantitation (LOQ) were determined experimentally. The criterion for acceptance of the LOQ value was a combined standard uncertainty of less than 10%. The accuracy of the method was checked by analyzing two matrix reference materials: ClinChek^®^ Whole Blood Control for Trace Elements, Level III (RECIPE, Munich, Germany) and Seronorm Trace Elements Whole Blood L-2 (SERO, Billingstad, Norway). Precision of measurement was ≤5% for all elements.

### 2.4. Statistical Analysis

Element concentrations in material from the medullary cavity or blood were expressed as mean ± standard deviation (SD). In addition, delta values (Δ) were calculated as the difference between post-operative and pre-operative measurements. Since the distribution of Δ values frequently deviated from normality, their descriptive statistics are presented as medians with interquartile ranges. The distribution of data was assessed using the Shapiro–Wilk test. For normally distributed variables with homogeneity of variances (tested by Levene’s test), paired *t*-tests were used to compare pre- and post-operative concentrations within the same tissue. For variables not meeting parametric assumptions, the Wilcoxon signed-rank test was applied for pre vs. post comparisons. For the analysis of delta values (Δ = post − pre), pairwise Wilcoxon signed-rank tests were performed to compare blood and material obtained from the radial and ulnar medullary cavities. *p*-values were adjusted for multiple comparisons using the Holm–Bonferroni correction. *p*-values < 0.05 were considered statistically significant. All univariate analyses were conducted using PQStat software, version 1.8.6 (PQStat Software, Poznań, Poland) and Statistica software, version 13.3 (TIBCO Software Inc., Palo Alto, CA, USA).

Multivariate analyses were conducted only for blood samples to explore the relationships between element concentrations. Heatmaps were generated on standardized data, and clustering was performed using Euclidean distance and Ward’s method. In addition, Principal Component Analysis (PCA) and Partial Least Squares Discriminant Analysis (PLS-DA) were applied to evaluate the separation of blood samples based on elemental composition. Multivariate analyses were carried out using ClustVis (Bioinformatics, Algorithms and Data Mining Group, University of Tartu, Estonia; https://biit.cs.ut.ee/clustvis/) [14].

## 3. Results

In the study, the levels of six elements were investigated, including the main components of the implants (titanium and aluminum) as well as the key elements involved in bone metabolism and healing (calcium, magnesium, copper, and zinc). Titanium and aluminum were measured to evaluate the potential release of metal ions from the implant surface and their systemic distribution. Calcium serves as the primary mineral component of bone, essential for proper mineralization, while magnesium supports enzymatic activity and regulates the mineralization process. Copper plays a crucial role in maintaining bone structure through collagen cross-linking, and zinc contributes to skeletal regeneration by stimulating osteoblast function and inhibiting osteoclast-mediated bone resorption [15].

### 3.1. Changes in Metal Ion Content in the Material from the Medullary Cavity and Blood

The content of Ca, Cu, Mg, Zn, Ti, and Al was analyzed in samples of the material from the medullary cavity and peripheral blood collected before fixation and at the time of plate removal. The results are summarized in Table 2.

No statistically significant changes were found for all investigated elements, except for titanium in blood samples, where a slight increase in concentration was observed. When comparing the mean values before and after, a statistically significant difference was detected (*p* = 0.0075).

**Figure 2:**
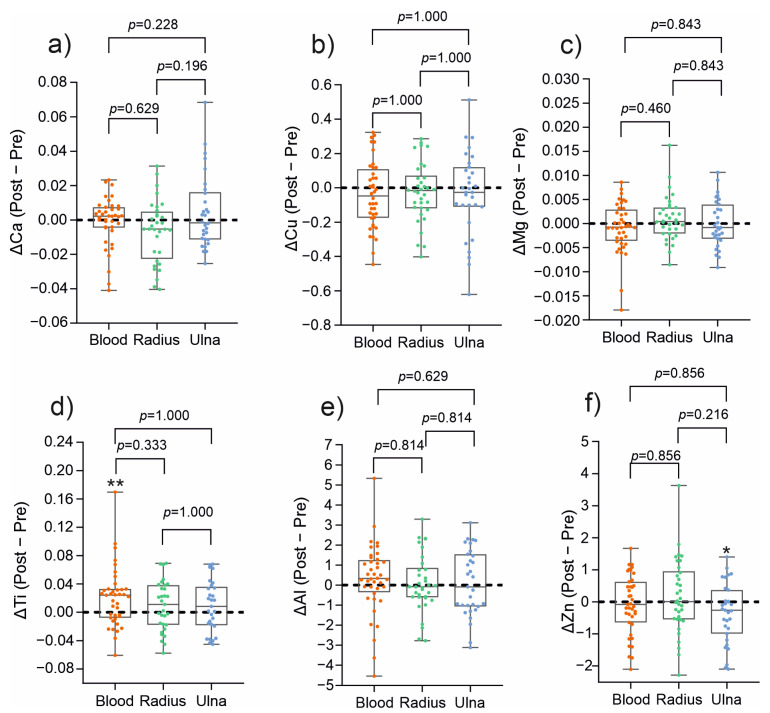
Comparison of delta values (Δ, post − pre) of (**a**) calcium (Ca), (**b**) copper (Cu), (**c**) magnesium (Mg), (**d**) titanium (Ti), (**e**) aluminum (Al), and (**f**) zinc (Zn) in blood, and in material obtained from the radial and ulnar medullary cavities. Data are presented as median (line), interquartile range (box), and minimum–maximum values (whiskers), with individual values shown as dots. Pairwise comparisons between tissues (blood vs. ulna, blood vs. radius, and ulna vs. radius) were performed using the Wilcoxon signed-rank test, and *p*-values were adjusted for multiple testing using the Holm–Bonferroni correction. Asterisks indicate significant pre- vs. post-operative differences within a tissue (* *p* < 0.05, ** *p* < 0.01).

To evaluate the true effect of treatment, elemental concentrations were expressed as delta values (Δ), representing the difference between post-treatment and pre-treatment measurements (Figure 2). This approach eliminates inter-individual variability in baseline concentrations, allowing for a direct assessment of treatment-related changes. The investigation revealed that the concentrations of all the analyzed metal ions were not different between blood, material obtained from the radial and ulnar medullary cavities, suggesting a comparable compartmental distribution. Single changes were detected only for two specific conditions: increase in Ti levels in blood after treatment, reflecting minor release of Ti from the implantation rods, and decrease in Zn material obtained from the ulnar medullary cavities. No variations were observed for all the remaining elements (Ca, Mg, Cu, Al). Clinically, the findings substantiate that titanium implantations do not elicit systemic disturbances of mineral homeostatic balance, and observed changes are limited to minor, local variations. The minor increase in Ti detectable in the circulation may be interpreted as a marker of exposure to the implant rather than of toxicity. At the same time, a local decrease in Zn in the material obtained from ulnar medullary cavities warrants further investigation but appears not to influence systemic balance.

**Figure 3:**
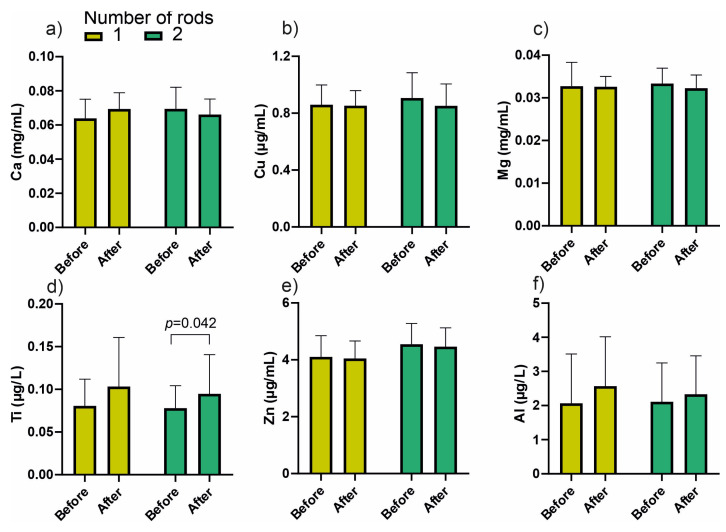
Concentrations of metal ions in blood samples collected before and after treatment, stratified by the number of implanted titanium rods (1 vs. 2). Elements presented: (**a**) calcium (Ca), (**b**) copper (Cu), (**c**) magnesium (Mg), (**d**) titanium (Ti), (**e**) zinc (Zn), and (**f**) aluminum (Al). Data are shown as mean ± SD.

In the next stage, an in-depth statistical analysis was carried out with division into groups including sex and, in the case of blood, the number of rods used for stabilization. No statistically significant differences were observed for the elemental content in material obtained from the medullary cavities. The results concerning blood, divided according to the number of rods, are presented in Figure 3.

A significant increase in blood titanium concentration was observed after treatment in patients with two rods (*p* = 0.042), while no significant differences were detected for other elements.

### 3.2. Multivariate Analysis of Metallic Elements Concentration in Blood Samples

In order to explore the global patterns of variation in metallic element concentrations in blood samples collected before and after treatment, multivariate statistical tools were applied. Principal component analysis (PCA) revealed that the first two components explained 49.1% and 24.0% of the total variance, respectively (Supplementary Figure S1). Copper, Mg, and to some extent Al showed strong loadings on PC1, suggesting that these elements varied together and represented a common source of variance. In contrast, Ti, and to some extent Ca, together with Zn contributed most strongly to PC2, but with opposite signs (Ti and Ca vs. Zn), indicating that Zn was negatively correlated with Ti and Ca. When plotting individuals on the PCA score plot, pre- and post-treatment samples showed substantial overlap. This indicates that the overall metallic element profiles remained largely similar following treatment and that no clear separation between groups could be established. Although Ti and Zn explained much of the variation captured by PC2, this axis did not discriminate between pre- and post-treatment states, but rather reflected inter-individual variability.

**Figure 4:**
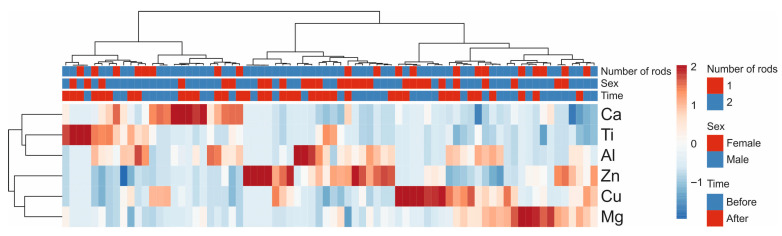
Heatmap with hierarchical clustering of standardized metallic element concentrations in blood. Rows correspond to individual elements and columns to patients. Annotation bars indicate the number of fractured bones (1 vs. 2), sex (male vs. female), and the time point of measurement (before vs. after treatment). The color scale represents relative element abundance (blue = lower, red = higher).

The relationship between elements was confirmed by heatmap analysis (Figure 4). Hierarchical clustering based on standardized data showed that Ti and Ca, together with Al, tended to cluster closely, indicating related patterns of variation across patients. In contrast, Zn formed a distinct branch, clearly separating from the Ca-Ti-Al cluster. Cu and Mg were grouped into another cluster, reflecting unique but partially related variability. Importantly, the heatmap analysis did not reveal any clear impact of treatment status (before vs. after), sex, or the number of rods, suggesting that these clinical variables did not drive the observed clustering patterns.

**Figure 5:**
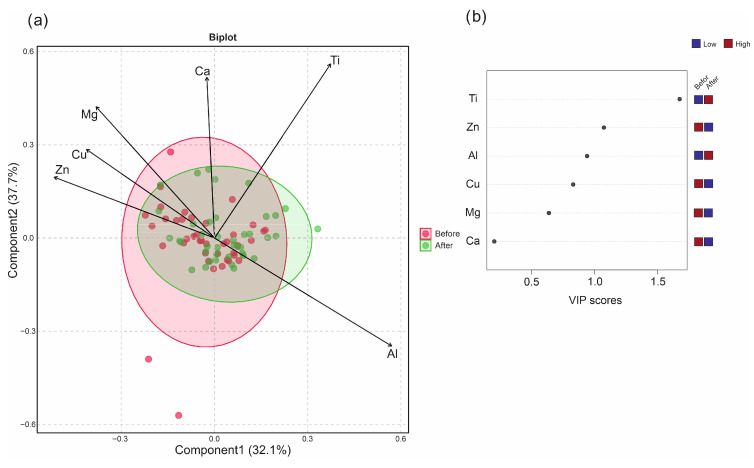
Differentiation of blood samples collected before and after treatment. (**a**) Partial least squares discriminant analysis (PLS-DA) of metallic element concentrations in blood samples collected before (red) and after (green) treatment. (**b**) Variable importance in projection (VIP) scores of samples.

In order to evaluate potential discriminative patterns, PLS-DA was performed (Figure 5). Although the cross-validation statistics (accuracy~0.58, R^2^~0.10, Q^2^~0.02) indicated limited predictive power, the model suggested that Ti contributed most strongly to the separation between groups. Specifically, post-treatment samples showed slightly higher Ti levels in blood compared to pre-treatment. This observation may represent a subtle but biologically plausible effect of titanium rod implantation on circulating Ti concentrations. Thus, while the global element profile remained largely stable, the consistent contribution of Ti in the multivariate analyses points toward a minor treatment-related influence. Taken together, the multivariate analyses confirmed that the overall elemental pattern remained stable after treatment, with titanium being the only variable showing a consistent, though small, post-treatment increase.

## 4. Discussion

The use of orthopedic implants in fracture treatment has expanded the possibilities for correction and improved outcomes, while also reducing the risk of treatment failure by providing additional stabilization of the fracture. However, it may also be associated with complications and disorders resulting from implantation and from leaving the fixation in place temporarily or permanently. The growing prevalence of bone fixation devices in fracture management raises important questions about their systemic effects on the body. In recent years, complications caused by metal ions, residual metal particles, and organometallic compounds in orthopedic patients have become an increasingly significant concern [16,17]. Both local and systemic toxicity induced by products released from implants, which may also disrupt elemental homeostasis, should be carefully considered.

In our study, disturbances in calcium (Ca), copper (Cu), magnesium (Mg), and zinc (Zn) levels were investigated in children following stabilization of forearm fractures with implants, as these elements are considered critical for bone metabolism, fracture healing, and possible implant–tissue interactions. Calcium, magnesium, and zinc are required for bone mineralization, callus formation, and the progression of fracture healing, whereas copper is involved in enzymatic processes necessary for collagen cross-linking and connective tissue repair [18]. In addition, titanium and aluminum have been monitored, as they may be released from orthopedic implants through corrosion or mechanical wear, and tracking their levels is recommended to evaluate potential biocompatibility concerns [19]. Our study showed that there were no significant disturbances in the levels of calcium, copper, magnesium, or zinc, either in peripheral blood or in material obtained from the radial and ulnar medullary cavities, in children following fracture stabilization. Although a slight upward trend in titanium concentration in the blood was observed during follow-up, the changes were mild, and the levels of Ti and Al did not exceed the values given in the literature [20,21,22,23]. For example, Li et al. reported that normal titanium concentrations in healthy pediatric populations range from 0 to 1 ng/mL [24]. The present findings are consistent with these reports, confirming the safety of this treatment method in pediatric patients and supporting the continued use of modern orthopedic implants as a reliable option for fracture stabilization. The observed increase in titanium, therefore, reflects minimal systemic exposure associated with the presence of titanium implants rather than any adverse biological effect.

There is limited literature on metal ion release in pediatric patients, as most studies focus on adults, particularly those with permanent implants [25,26,27,28]. The majority of research has examined titanium, molybdenum, and cobalt, which are the primary metals used in orthopedic implant alloys [25,29,30]. Regarding children, disturbances in metal levels have been studied in the context of spinal deformity correction using different implants. Cundy et al. reported two-year results for five spinal instrumentation systems, namely URS System, Expedium Spine System, Expedium Verse Spinal System, Mesa Deformity Spinal System (K2M/Stryker), and Response Spine System with varying metal compositions. At two years, titanium was 5.14 times higher and cobalt was 1.74 times higher compared with pre-surgery control levels [31]. In another study of children after instrumented spinal arthrodesis (Ti6-Al7-Nb instrumentation), increased titanium and Nb levels were also observed throughout the first year postoperatively [32]. Alberghina et al. similarly found elevated titanium levels in 87% of patients treated for early-onset scoliosis using magnetically controlled growing rods (MCGR), with an average treatment duration of 4.8 years [33]. Mathew et al. noted higher Ti, Co, and Cr levels in patients with spinal implants, including MCGR, traditional growing rods, and vertical expandable prosthetic titanium rib (VEPTR), particularly in children with the MCGR system [34]. Danielewicz et al. found elevated Ti levels in surrounding tissues and blood, though within permissible limits, in patients treated with the Traditional Growing Rod (TGR), Guided Growth System (GGS), and VEPTR systems during a mean follow-up period of 2 ± 2.8 years [21]. Badhe reported elevated metal ion levels following orthopedic implant use, with no systemic toxicity, though local metal toxicity was observed, potentially necessitating implant revision [17]. In contrast, McGarry found no increase in serum titanium or any differences in metal ion levels during the first year after femoral nailing with titanium alloy (Ti-6Al-7Nb) [35].

Interestingly, several studies suggest that elevated metal levels may also originate from surgical instruments used during the procedure. Pediatric spine surgery studies have demonstrated an immediate postoperative peak in most serum metal ion levels [32,36]. An increase in metal ion concentrations has also been confirmed in adult patients. For example, Hanawa et al. reported that metal ions are released from stainless steel (Fe–Cr–Ni alloys), cobalt–chromium (Co–Cr–Mo alloys), and titanium (Ti–6Al–4V) implants used for long-bone fracture stabilization and prosthetic procedures [29]. Similarly, Tonoglu et al. observed significant postoperative increases in Ti, Mo, Al, and V levels in adult patients following internal stabilization of lower-limb fractures with a titanium alloy (Ti-6Al-4V) over a 24-week period [37]. In turn, Patton et al. reported increased metal ion concentrations in patients treated with intramedullary nails made of stainless steel (316 LVM) (mean in situ duration: 26 months) or titanium alloy (Ti-6Al-4V) (mean in situ duration: 43 months) compared to controls. Stainless steel nails led to a nearly 2.5-fold rise in chromium, while titanium nails increased serum titanium levels [38]. Sansone et al. demonstrated elevated metal ion levels in bodily fluids and tissues of patients after hip and knee arthroplasty (Ti alloys, Co-Cr-Mo alloys, and stainless steel), with associated osteolysis around implants [39]. Savarino et al. confirmed increased nickel and chromium levels in patients with stainless steel orthopedic implants (four plates and five nails) [30], and Witzleb et al. observed elevated chromium and cobalt after hip arthroplasty using BHR (Birmingham Hip Resurfacing) and TiAl6Nb7 alloy, with molybdenum remaining unchanged [25]. Jiang reported significant serum increases in Cr, Co, and Mo after hip resurfacing arthroplasty [40]. Sargeant and Goswami reported elevated levels of Ti, Al, V, Cr, Co, and Mo with potential carcinogenic implications in their review article [41].

As can be seen, in contrast to our investigation, most studies have reported a significant increase in metal ion levels. However, it should be noted that the duration of implant retention in those studies was considerably longer than that typically observed in pediatric patients following fracture stabilization (averaging about six months).

Taken together, these data indicate that metal ion release from orthopedic implants is a clinically relevant issue, highlighting the need for systematic monitoring, long-term studies, and careful evaluation of potential local and systemic effects. As demonstrated by our study, in the case of forearm fracture stabilization in children, where the duration of implant retention is relatively short, there are no significant disturbances in elemental homeostasis, and the concentrations of potentially toxic elements present in implant alloys do not increase to clinically relevant levels.

The study had certain limitations, including a relatively small patient cohort, which suggests the need for further research involving larger populations. Furthermore, metal ion measurements were performed only in blood, which may not fully reflect their distribution in other tissues or body fluids. Future studies could benefit from a broader assessment, including additional elements and other biological compartments, to provide a more comprehensive understanding of metal exposure and its potential effects. Moreover, the study focused exclusively on forearm fractures, as these are significantly more common in children, which allowed efficient recruitment and a statistically valid sample size. However, this specificity may limit the generalizability of the findings to fractures in other anatomical locations or other fixation methods.

## 5. Conclusions

This study demonstrated that intramedullary fixation of forearm fractures in children does not significantly disturb systemic mineral homeostasis. Concentrations of calcium, magnesium, copper, and zinc remained stable, while only a mild, clinically insignificant increase in blood titanium levels was observed. Post-operative titanium concentrations stayed within the physiological range for healthy pediatric populations and well below toxicity thresholds, confirming the safety and biocompatibility of titanium implants. Unlike studies in adults, where longer implant retention often leads to measurable increases in circulating metal ions, the relatively short duration of fixation in pediatric patients appears to prevent significant systemic metal release. These findings support the continued use of modern titanium-based implants as a safe and reliable option for fracture management in children, while emphasizing the need for long-term monitoring and larger cohort studies to further assess potential cumulative or subclinical effects.

## Figures and Tables

**Table 1:** Detailed data of patients included in the study.

Characteristic	Total (n = 40)	Girls (n = 14)	Boys (n = 26)
Sex, n (%)		14 (35%)	26 (65%)
Age at first surgery, years (mean ± SD)	9.7 ± 3.1		
Distribution of fracture types among patients			
Ulna fracture only	4	1	3
Radius fracture only	8	2	6
Both bones	28	11	17

**Table 2:** Concentration of elements Ca (mg/mL), Cu (µg/mL), Mg (mg/mL), Zn (µg/mL), Ti (µg/L), Al (µg/L) in the blood and material obtained from the radial and ulnar medullary cavities before and after intramedullary fixation.

Element	Stabilized Ulna			Stabilized Radius				Blood	
	Before	After	*p*-Value	Before	After	*p*-Value	Before	After	*p*-Value
Ca	0.067 ± 0.001	0.071 ± 0.018	0.282	0.077 ± 0.003	0.069 ± 0.009	0.056	0.068 ± 0.012	0.067 ± 0.009	0.713
Cu	0.866 ± 0.026	0.831 ± 0.030	0.434	0.856 ± 0.144	0.830 ± 0.121	0.395	0.892 ± 0.166	0.852 ± 0.140	0.198
Mg	0.032 ± 0.003	0.032 ± 0.004	0.972	0.031 ± 0.004	0.032 ± 0.003	0.223	0.033 ± 0.004	0.032 ± 0.003	0.332
Zn	4.476 ± 0.734	4.135 ± 0.570	0.049	4.026 ± 0.786	4.187 ± 0.803	0.422	4.408 ± 0.748	4.335 ± 0.667	0.621
Ti	0.089 ± 0.041	0.091 ± 0.035	0.764	0.079 ± 0.023	0.087 ± 0.027	0.170	0.079 ± 0.028	0.097 ± 0.487	0.006
Al	2.354 ± 1.235	2.466 ± 0.198	0.709	2.242 ± 1.223	2.327 ± 1.088	0.732	2.099 ± 1.220	2.402 ± 1.218	0.278

## Data Availability

The data presented in this study are available on request from the corresponding author.

## Supplementary Materials

The following supporting information can be downloaded at: https://www.mdpi.com/article/10.3390/jcm14217829/s1, Figure S1: Principal Component Analysis (PCA) biplot of metallic element concentrations (Ca, Cu, Mg, Ti, Zn, Al) in blood samples before (red) and after (green) surgery.
