# Supplementary material for: Impact of Intramedullary Implants on Metallic Element Homeostasis in Children with Forearm Fractures
Source: J Clin Med. 2025 Nov 4;14(21):7829. doi: 10.3390/jcm14217829 (PMC12610260; doi:10.3390/jcm14217829)
Supplement: Supplementary file 1 [file jcm-14-07829-s001.zip › jcm-3955473-supplementary.pdf]

Supplementary

Impact of Intramedullary Implants on Metallic Element Homeostasis in Children with Forearm Fractures

Kacper Sowa [1], Anna Danielewicz [1], Magdalena Wójciak [2], Jan Sawicki [2], Sławomir Dresler [2], Katarzyna Warda [1], Michał Latalski [1,*] and Ireneusz Sowa [2,*]

1 Department of Paediatric Orthopaedics, Medical University of Lublin, 20-059 Lublin, Poland; kacper.sowa@umlub.edu.pl (K.S.); anna.danielewicz@umlub.edu.pl (A.D.); katarzyna.m.warda@gmail.com (K.W.)

2 Department of Analytical Chemistry, Medical University of Lublin, 20-059 Lublin, Poland; magdalena.wojciak@umlub.edu.pl (M.W.); jan.sawicki@umlub.edu.pl (J.S.); slawomir.dresler@umlub.edu.pl (S.D.)

* Correspondence: michallatalski@umlub.pl (M.L.); ireneusz.sowa@umlub.edu.pl (I.S.)

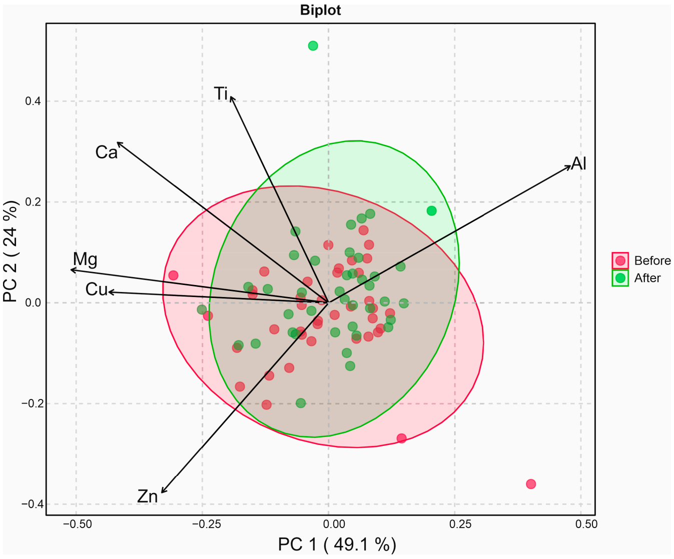

Figure S1. Principal Component Analysis (PCA) biplot of metallic element concentrations (Ca, Cu, Mg, Ti, Zn, Al) in blood samples before (red) and after (green) surgery.
